# Comparative evaluation of maintenance of cell viability of an experimental transport media “coconut water” with Hank's balanced salt solution and milk, for transportation of an avulsed tooth: An *in vitro* cell culture study

**DOI:** 10.4103/0972-0707.43414

**Published:** 2008

**Authors:** Toby Thomas, Velayutham Gopikrishna, Deivanayagam Kandaswamy

**Affiliations:** Department of Conservative Dentistry and Endodontics, Meenakshi Ammal Dental College and Hospitals, Madhuravoyal, Chennai, India

**Keywords:** Avulsed teeth, coconut water, storage medium

## Abstract

The purpose of this study was to evaluate the efficiency of a new storage medium, coconut water, in comparison with other traditional storage media like Hank's balanced salt solution (HBBS) and milk, in maintaining the viability of an established cell line BHK-21/C13 (baby hamster kidney fibroblasts) using the direct suspension cell culture technique.

The storage media tested in the study were divided into three major groups and two control groups - Group A: HBBS, Group B: milk, and Group C: coconut water. The positive and negative controls corresponded to 0-minute and 24-hour dry times respectively.

The three groups were then divided into five subgroups, each denoting the storage time periods 15 min, 30 min, 45 min, 60 min and 120 min respectively. The cell line BHK-21/C13 was subcultured and the number of cells was standardized by making a cell suspension using Minimal Essential Medium in five culture plates.

One ml of each experimental group (HBBS, milk and coconut water) was added to eight wells of each culture plate. The culture plates containing the cells and the experimental groups were incubated for the respective time periods. The cells were then counted with a Neubauer counting chamber, under light microscope. The results were statistically analyzed using One-way ANOVA and Multiple Range Test using the Tukey-HSD procedure to identify the significant groups at p ≤ 0.05.

Within the parameters of this study, it appears that coconut water may be a better alternative to HBSS or milk, in terms of maintaining cell viability. Coconut water can be used as a superior transport medium for avulsed teeth.

## INTRODUCTION

Clinical surveys indicate that traumatic dental injuries in children and adolescents are a common problem. Studies have shown that the prevalence of these injuries are increasing.[1] Avulsion injury, one of the most severe forms of dental trauma, is characterized by complete displacement of the tooth from its alveolar socket. Due to the complexity of this injury, the neurovascular supply is severely compromised and usually results in loss of pulp vitality.

In cases of tooth avulsions, the primary goal is to preserve the vitality of the periodontal ligament (PDL) cells attached to the root surface, until appropriate treatment can be performed. This may bring about a favorable reattachment of the periodontal ligament. Soder *et al.*[2] showed that after avulsion, the number of viable cells on the root surface decreased with increased drying time and that after two hours, it would not be possible to demonstrate cell viability. Therefore, the ideal treatment of choice at the time of avulsion should be immediate replantation, in order to reestablish the natural nutrient supply to the periodontal ligament cells. This would also minimize further damage and enhance the healing process. Unfortunately, there may be situations where there may be a delay in replantation. Under such circumstances, the tooth should be stored in a medium that maintains periodontal ligament cell viability, until a definitive dental treatment can be accomplished.

The ability of a storage/transport medium to support cell viability can be more important than the extra oral time, for preventing ankylosis and replacement resorption.[2–5] Various storage media such as tap water, saliva, saline, milk, culture media, and Viaspan have been investigated for their ability to maintain cell viability.

Krasner and Person[6] introduced a commercially available form of Hank's Balanced Salt Solution (HBSS), marketed as Save-A-Tooth, which was proven to maintain periodontal ligament cell viability. Unfortunately, HBBS is not widely used in India, because it is not readily available to the public. Hence, an alternative medium that is not only comparable in performance to HBBS, but is also readily available and inexpensive is required.

Tender coconut water is technically the liquid endosperm of the coconut. It is the safest natural soft drink, with no detrimental outcomes. The biologically pure, tender coconut water helps to replace fluids and electrolytes (potassium, sodium and chloride) and sugars lost from the body during heavy physical exercise. It is used as a blood plasma substitute as it is sterile, does not produce heat, does not destroy red blood cells and is readily accepted by the body.[7] Taking these properties into consideration, we hypothesized that this natural isotonic drink could be a viable storage medium for the transportation of avulsed tooth.

The use of coconut water as a storage medium for avulsed tooth was recently proposed by Gopikrishna *et al.*[8,9] However, no study till date has compared the efficacy of coconut water as compared to milk. This is the first study to employ cell culture study, using established cell line BHK-21/C13, for the assessment of coconut water as an efficient storage medium. Hence, the purpose of this study was to evaluate the efficiency of a new storage medium, coconut water, in comparison with other traditional storage media such as HBBS and milk in maintaining the viability of an established cell line BHK-21/C13, using the direct suspension cell culture technique.

## MATERIALS AND METHODS

The storage media tested in the study were divided into three major groups:-

Group A: Hank's Balanced Salt Solution

Group B: Milk

Group C: Coconut water

The three groups were then divided into five subgroups, each denoting the storage time periods 15 min, 30 min, 45 min, 60 min and 120 min respectively.

### Positive control

80,000 cells/ml were counted soon after the formation of a monolayer.

### Negative control

No medium was used and the cells were left dry for 24 hours.

To standardize the number of cells, a cell suspension was made to get a cell count of 40,000 cells/ml, using the Minimal Essential Medium (MEM). The cells were counted with a Neubauer counting chamber, under light microscope (as WBC count).

In this study, five culture plates, each having 24 wells, were used. From the cell suspension, 1 ml was distributed to each well of the culture plate. Likewise, five culture plates were created and placed in an incubator containing 5% CO_2_ at 37°C for 48 hours, to form a complete monolayer.

After 48 hours, the MEM was discarded using a pipette and 1 ml of each experimental group, namely HBBS, milk and coconut water were added to eight wells of each culture plate. Each experimental group was allotted eight wells for a time period of 15 min, 30 min, 45 min, 60min and 120 min. A total of five culture plates were used, one for each time period. The culture plates containing the cells and the experimental groups were incubated for 15 min, 30 min, 45 min, 60 min and 120 min respectively.

In the case of every group, after the completion of the test period, the experimental medium was discarded from each well of the culture plate, using a pipette, and 0.5ml of 0.1% TVG was added and left for 30 seconds to detach the cells from the bottom of the well. The TVG was then discarded and its action was stopped by adding 1 ml of fresh medium MEM containing 10% fetal bovine serum. 1 ml of 0.25% wt/vol Trypan blue solution was taken in a pellet, to which 1 ml of the cell suspension was added. The cell suspension and the Trypan blue were mixed well by gentle pipetting within the pellet, using a micropipette. and then the suspension was placed on a Neubauer counting chamber for analyzing the number of viable and nonviable cells under an inverted light microscope. (The cell counting is done as described earlier).

**The cell concentration per ml is calculated as:-**

**Cell count^∗^** × **2 (if Trypan blue is used)** × **104**

^∗^ The count in one square or an average of a count if more than one square is used.

**The percentage of viable cells is calculated by the formula:-**

$$\mathrm{Percentage of viable cells}=\frac{A}{B}\times 100$$

A: number of viable cells in the experimental wells

B: number of viable cells in the control wells

This procedure was repeated for the other subgroups also.

Mean and standard deviation were estimated from each group for each time period. Mean values were compared by One-way ANOVA. Multiple Range Test by Tukey-HSD procedure was employed to identify the significant groups, if *P* - value in One-way ANOVA was significant.

In the present study, *P* ≤ 0.05 was considered as the level of significance.

## RESULTS

At 15 minutes, among the three Groups, Group A (Hank's Balanced Salt Solution) had significantly higher number of viable cells, as compared to that of Group B (Milk) and Group C (Coconut Water) [Table 1].

**Table 1:** Average number of cells/ml counted in different groups at different time periods

Time periods	Group A (Milk)	Group B HBSS	Group C (Coconut water)
15 min	67,500	42,500	52,500
30 min	55,000	47,500	50,000
45 min	50,000	37,500	42,500
60 min	51,300	40,000	42,500
120 min	47,500	32,500	37,500

At 30 min, 45 min and 60 min, there was no statistically significant difference in the number of viable cells among the three groups [Table 2].

**Table 2:** Mean, standard deviation and test of significances of mean percentage values between different groups at different time periods

Time (in min)	Group	Mean±SD	*P*- Value	Significance at *P*≤ 0.05
15	HBSS	84.4±18.6	0.002(Sig)	A *Vs* B
	Milk	56.3±11.6		A *Vs* C
	Coconut Water	59.4±12.9		

30	HBSS	68.8±11.6	0.53(Ns)	Nil
	Milk	59.4±18.6		
	Coconut Water	62.5±18.9		

45	HBSS	62.5±13.4	0.14(Ns)	Nil
	Milk	46.9±16.0		
	Coconut Water	53.1±16.0		

60	HBSS	64.1±18.2	0.13(Ns)	Nil
	Milk	50.0±13.4		
	Coconut Water	53.1±8.8		

120	HBSS	59.4±18.6	0.05(Sig)	A *Vs* B
	Milk	38.6±12.9		
	Coconut Water	46.9±16.0		

⋆ One - Way ANOVA was used to calculate the *P*- value.

⋆ Multiple range test using Tukey -HSD procedure was employed to identify the significant groups at *P*≤ 0.05

⋆ Sig - Significant, Ns - Non-significant.

At 120 min, there was a statistically significant difference in the number of viable cells between Group A (HBBS) and Group B (milk)

## DISCUSSION

Avulsion is a traumatic injury leading to loss of attachment of periodontal ligament from the alveolar socket. The treatment of choice, in such cases, is to replant the tooth into the socket. It has been shown that the most critical factor for successful replantation is the maintenance of viable periodontal ligament cells on the root surface.

One of the sequelae following replantation of avulsed tooth includes inflammatory or replacement resorption.[10] The development of inflammatory root resorption is directly related to damage of the periodontium at the time of accident and the presence of bacteria within the root canals and dentinal tubules.[3,11–16] The development of replacement resorption depends on both the degree of damage to the periodontium at the time of avulsion[12,16,17] and the extent to which the viability of periodontal ligament cells remaining on the tooth surface is maintained.[13] Hence, the prognosis of an avulsed tooth is largely dependent on the status of the periodontal ligament cells at replantation. Therefore, the predominant philosophy derived from the research of Andreasen and Hjorting – Hansen,[10,19] for the treatment of avulsed teeth, is: *Replant the tooth immediately or as quickly as possible after the avulsion*. But certain situations, such as the presence of more severe injuries needing immediate medical attention or nonavailability of a dental clinic close by, can lead to a delay in immediate replantation of the avulsed tooth. In such situations, the teeth should be stored in a medium that can maintain the periodontal ligament cell viability, until definite dental treatment is accomplished

Studies confirm that root resorption is a frequent complication in replanted teeth and that two of the most critical factors affecting the prognosis of an avulsed tooth are: extra oral dry time and storage medium in which the tooth is placed before replantation.[2,19] According to Andreasen and Hjorting-Hansen,[10,19] teeth that are replanted quickly (within 30 minutes) have a better success rate than those that were extra oral for longer periods of time before replantation.[10,18] Some experimental studies have indicated that storage medium is a more critical prognostic factor than the extra-alveolar period.[2,4,5,10] Physiologic storage media such as milk, saliva, saline, HBBS and Viaspan have been used for preserving the viability of periodontal ligament cells.[20–22]

Tap water, when used as a storage medium, was found to be unsuitable due to its hypotonicity leading to rapid cell lysis.[23] Extended extra oral storage in tap water showed a decrease in periodontal ligament cell viability[2,13] and increased external root resorption.[24,25]

Storage medium such as saliva was found to be more effective than tap water, but it also has a potential for bacterial contamination.[26] The osmolarity of saliva (60- 80mOsm/l) was found to be much lower than the normal range (230-400 mOsm/l required for cell growth.[27] Further, saliva is a hypotonic solution, causing periodontal ligament cells to swell and burst.[28]

Blomlof *et al.*[13,17] has shown storage of avulsed teeth in tap water and saliva to be damaging to periodontal ligament cells, causing increased root resorption.[13,17,24,25] Saline has been found to be a short term storage medium because of its physiologic osmolality.[29] Cvek *et al.*[14] found that avulsed teeth that were soaked in an isotonic saline solution for 30 minutes before replantation showed less resorption then those that were stored dry for 15 and 40 minutes.

Viaspan, a new storage medium presently used for organ transplant storage,[20] has proved to be an extremely effective cold storage medium for organs before replantation.[30,31] While its effectiveness is not completely explained, the impermeants lactobionate and raffinose are thought to prevent cell swelling, which is an important factor in maintaining the vitality of the cells. Also, it has an effective hydrogen ion buffer (Disodium hydrogen phosphate), which might be important in maintaining the pH. It also contains adenosine, which is necessary for of cell division.[32] Both HBBS and Viaspan proved to be superior to milk, with Viaspan clearly indicating the most effective medium with 37.6% vital fibroblasts after 168 hours of storage, thus showing a potential value as a superior long-term storage medium.[20]

Recent studies have evaluated the use of 0.9% isotonic saline, milk, HBBS and Viaspan as storage media for the preservation of cell viability.[20–22] Hank's Balanced Salt Solution was the most effective,[20,22] although milk and saline were suitable, provided the extra oral time did not exceed two hours.[21]

Practically speaking, HBBS is not commonly available to a majority of the people at the time of an accident; hence, milk has been advocated as an appropriate storage medium,[4,13,21,33–36] as it was found to maintain the viability of periodontal ligament cells.

Apart from maintaining the viability, a medium should be easily available, inexpensive and simple to use. Tender coconut water has been proved to be a blood plasma substitute because of its sterility and biocompatibility.[7] In the Indian scenario, coconuts are more easily available, as compared to the above mentioned medium (Hank's Salt Solution). India is also the largest coconut producing country in the world, with an annual production of 12,821 million nuts from an area of 18.92 lakh hectares.[37] Hence, we hypothesized employing coconut water as an experimental transport medium in this study.

Several techniques have been used to determine the viability of the periodontal cells following avulsion. However, most of the experimental data that is available has been obtained using techniques in which the cells are cultured and/or trypsinized stained and then counted with a haemocytometer. Though cell culture studies are nowhere equal to *in vivo* studies, a cell culture study has been chosen because one of the main problems in conducting an *in vivo* study is that the procedure is difficult to standardize. During extraction, the number of viable cells can be disturbed. Traumatic extraction can seriously affect the number of cells on the root surface; hence, it is difficult to quantify the number of viable cells in each tooth after extraction, which in turn leads to improper standardization of specimens. Whereas in a culture study, cells are made to grow using growth media and once the confluence of cells is obtained, the number of cells can be determined per ml and seeded in culture wells or a flask to which experimental groups can be added and tested. Hence, a much more accurate analysis is possible.

Established cell lines such as BHK-21/C13 (baby hamster kidney fibroblasts), L929 mouse fibroblasts, HeLa human cervical carcinoma epithelial cells and Vero Monkey Kidney cells are commonly used for endodontic research, since they are readily available, easily cultured and consistent in quality.[38,39] Hence, in our study an established cell line of BHK-21/C13 baby hamster kidney fibroblasts was subcultured and used in a density of 40,000 cells/ml.

The results in the study were statistically analyzed by multiple range test, using the Tukey-HSD procedure to identify significant group at *P* ≤ 0.05. Comparing the different groups at different time periods, it was found that at 15 minutes, HBBS was found to produce excellent cell viability, as compared to milk and coconut water; and at 120 minutes, HBBS performed better than milk, whereas coconut water was equal to HBBS.

On comparing the cells at different time periods within the same group, it was found that Group A (HBBS) showed a statistically significant decrease in the number of viable cells from 15 to120 minutes, but was equally good between 30 and 120 minutes.

In Group B (milk), it was found that there was a statistically significant decrease in the number of viable cells from 15-120 minute time periods and 30-120 min time periods.

In Group C (coconut water), it was found that there was no statistically significant decrease in the number of viable cells between 15 and 120 minutes and that it was equally good.

A commercially available tooth preserving system utilizing HBBS as a storage medium, Save-A-Tooth, has become available for storage of avulsed teeth until replantation. An added advantage of this system is an inner suspension netting and a removable basket which permits general washing and removal of the tooth, without crushing of the periodontal ligament by the dentist. In our study, freshly prepared HBBS has been used as the storage medium for evaluating the viability of BHK-21/C13 cells. HBBS also contains essential nutrients and buffering agents capable of maintaining the pH of the solution, which can be the reason for maintaining the viability of the cells.[32]

Hiltz and Trope,[20] in their study, have mentioned that HBBS is a culture medium with excellent capacity for maintaining the vitality of cells of periodontal ligament and that the cells stored in HBBS did not show any distortion of morphology. Besides, the cells were normal in appearance. They also found that HBBS could keep 70% of the fibroblast viable for 96 hours. Trope and Friedman,[22] in their study with HBBS, showed a dramatic drop in complications at 72 h and 96 h storage times and this strengthens the hypothesis that the condition of the socket as well as the viability of periodontal ligament cell on the root surface might be critical for successful replantation. Krasner and Person[6] and Trope[40] have suggested that 30 min in HBBS would improve the chances of success of avulsed teeth with less than 60 min extra oral dry time. Krasner and Person[6] also suggest that the rehydration step is helpful up to 120 min dry time.

In their study, Ashkenazi *et al.*[41] showed that HBBS is the most effective storage medium for preserving the viability, mitogenicity and clonogenic capacity of periodontal ligament cells, after storage up to 24 hours, at 22°C. It is also seen that the mitogenic capacity of periodontal ligament fibroblasts was higher when stored at 4°C. When compared to room temperature, this might be attributed to the priming effect of the low temperature of the stored cells.[42] Thus, the above discussion proves that HBBS is the most effective storage medium for preserving the vitality of periodontal ligament cells.

**Figure 1:**
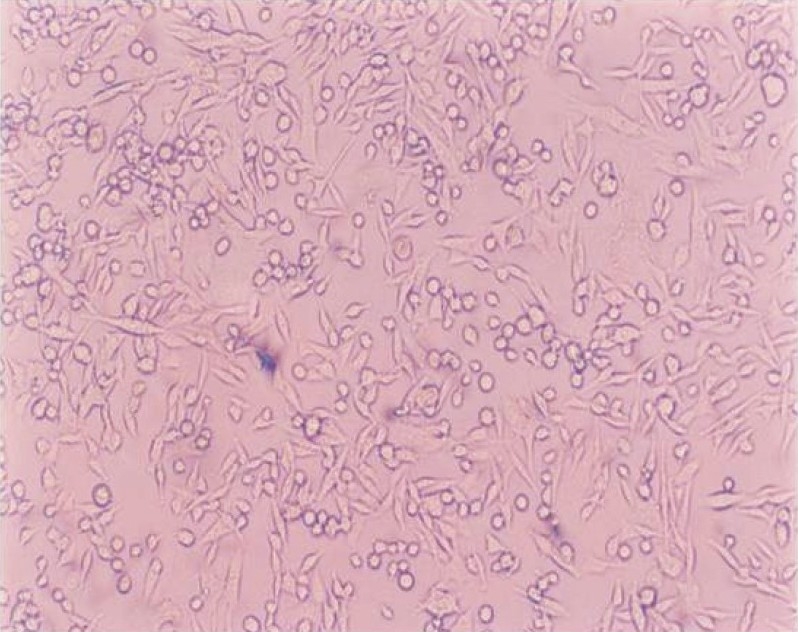
Hank's balanced salt solution

In this study, although HBBS showed excellent result at 15 min [Figure 1], clinically it is not possible, because the time taken to transport the teeth might be much longer. In addition, the disadvantage of HBBS is that it is not easily available to the common man; whereas, from 30 to 120 minutes, coconut water was found to be as good as HBBS. Also, coconut water, which is easily available to the public, would be the most appropriate medium to transport avulsed tooth within two hours.

Milk has been seen to be a compatible short-term storage medium of avulsed teeth, when the teeth are placed in it within 15 to 20 minutes.[22] Milk only prevents cell death rather than restoring normal morphology and ability to differentiate and mitose. Gamsen *et al.*[43] showed that milk is able to maintain the osmotic pressure for periodontal ligament cells but it does not have the ability to reconstitute depleted cell metabolites and restore viability. Blomlof *et al.*[13,28] found that milk was a compatible storage medium for periodontal ligament cells, only when it was cold and fresh.

**Figure 2:**
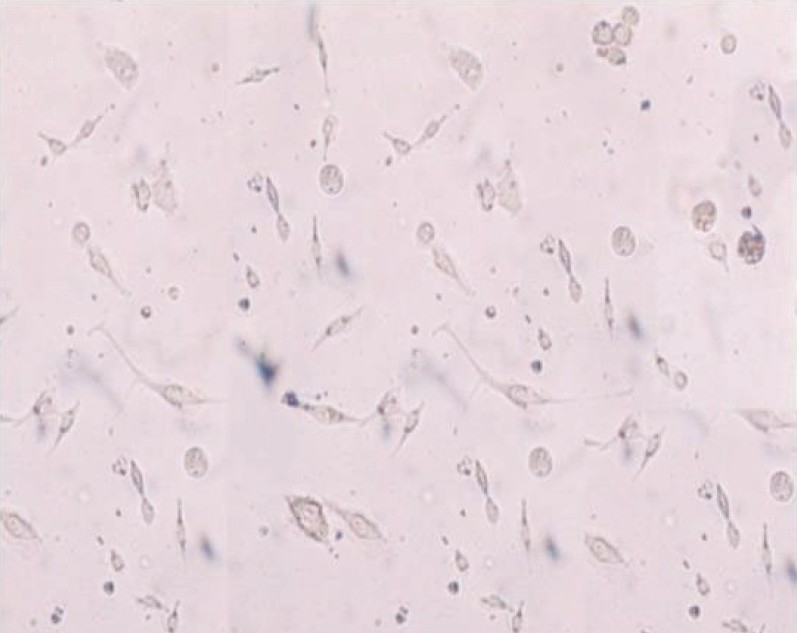
Milk

Hiltz and Trope[20] showed that fibroblasts stored in milk remained vital but their morphology was distorted. They speculated that the mitotic capacity of the cells would be diminished. Courts *et al.*[44] showed that periodontal ligament cells stored in milk were less capable of differentiating and that fewer cells were vital as compared to the cells stored in HBBS. There were 50% more vital periodontal ligament cells, when stored in HBBS than when stored in milk. Correlating with the results of our study, it proved that HBBS was better than milk at 120 min [Figure 2].

It has been recommended that even if avulsed tooth has been stored in physiologic media like saline and milk from the moment of the avulsion accident, the teeth should be still be soaked in HBBS for 30 min before replantation, because neither saline nor milk can replenish depleted cell metabolites.[45] The cells that are stored in milk are alive and unswollen, but they lack the cell energy and ions to permit the repopulation of the periodontal ligament. In addition, teeth stored in sour milk showed a decrease in periodontal ligament cell viability.[13]

**Figure 3:**
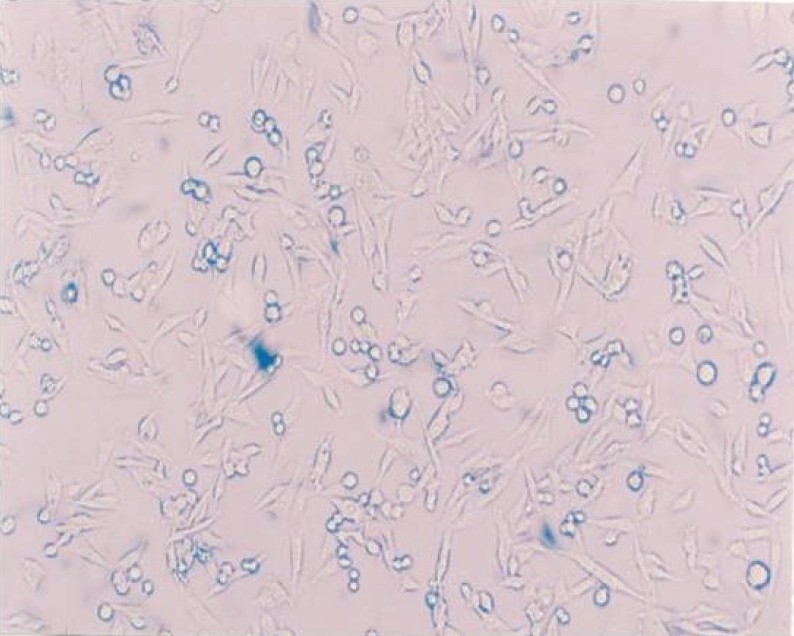
Coconut water

In our study, coconut water was found to keep up the viability as well as HBBS, from 30 min to 120 min [Figure 3]. Maintenance of viability of the cells may be due to the nutrients that are present in coconut water, such as proteins, amino acids, vitamins and minerals, which help in nourishing the cells and maintaining its viability. In a study conducted by Majunder,[46] coconut water was found to be sterile and nonhaemolytic. Ediriweera,[7] in his study, used coconut water intravenously as a substitute for normal saline. Hence, it can be hypothesized that as coconut water does not produce heat and destroy the RBCs, and given the fact that it is used as a blood plasma substitute, it can be a safer medium for the cells to be stored in and it can help maintain its viability. Quimo[47] reported that coconut water has auxinic or growth promoting properties that is liberally used in tissue culture techniques. This might be the reason why coconut water has enabled the cells to attach themselves to culture wells to form a monolayer; the mitotic and clonogenic activity of coconut water might have enabled it to maintain the viability of cells up to a period of two hours.

In their study, Gopikrishna and Toby[8] used a Collagenase-Dispase assay to investigate the potential of coconut water in maintaining viable periodontal ligament cells on simulated avulsed teeth, as compared to HBBS and milk. We found that coconut water kept significantly more PDL cells viable, compared to HBSS or milk.

Gopikrishna *et al.*,[9] compared the efficacy of coconut water with propolis, HBSS and milk in maintaining viable periodontal ligament cells on simulated avulsed teeth using Collagenase-Dispase assay. They found that coconut water had significantly more PDL cells viable compared with propolis, HBSS, or milk.

Blomlof[13] showed that the important factor in maintaining the viability is the osmolarity of the transport media. It has been reported that cell growth can occur at a range of 230-400mOsm/l.[27] When measured in an osmometer, the osmolarity of the HBSS, milk and coconut water was found to be 295mOm/l, 232mOsm/l, and 372mosm/l respectively. The osmolarity of HBSS was found to be well within the range, which has enabled it to keep up the viability of the cells for a period of two hours. Blomlof[13] found that HBSS was slightly better than milk, saliva or saline in maintaining the cell integrity. This was mainly due to its physiologic osmolarity. Milk was found to have the lowest osmolarity among the three (though it was still within the range), but the inability of the cells to multiply and divide might be the reason for a drastic decrease in number of viable cells from 15-120 min. The osmalarity of coconut water was found to be to be the highest, which might have enabled the cells to remain vital from 15-120 minutes without much of cell death.

This study was done mainly to check the viability of the cells and the results indicate that at 15 min HBBS is an excellent storage medium, after which coconut water can be thought of an appropriate storage medium. Further studies have to be done on the mitogenic and clonogenic capacity of coconut water, in comparison with HBBS, to measure its efficiency.

## CONCLUSIONS

Within the limitations of this *in vitro* study, the following conclusions are drawn:

1. Within 15 minutes, Hank's Balanced Salt Solution (HBBS) is the most effective storage medium.
2. After 15 minutes to 120 minutes, coconut water is as effective as HBBS.
3. The viability of cells in milk after one hour was statistically inferior to that of HBBS.
4. Due to the superior osmolarity, easier availability and cost effectiveness, coconut water can be advocated as a viable storage medium.
